# Capturing the Severity and Impairment Associated With Depression: The Overall Depression Severity and Impairment Scale (ODSIS) Validation in a Spanish Clinical Sample

**DOI:** 10.3389/fpsyt.2019.00180

**Published:** 2019-04-09

**Authors:** Adriana Mira, Alberto González-Robles, Guadalupe Molinari, Clara Miguel, Amanda Díaz-García, Juana Bretón-López, Azucena García-Palacios, Soledad Quero, Rosa Baños, Cristina Botella

**Affiliations:** ^1^Department of Psychology and Sociology, Universidad de Zaragoza, Teruel, Spain; ^2^Department of Basic and Clinical Psychology, and Psychobiology, Universitat Jaume I, Castelló de La Plana, Spain; ^3^CIBER Fisiopatología Obesidad y Nutrición, Instituto Salud Carlos III, Madrid, Spain; ^4^Department of Personality, Evaluation and Psychological Treatment, Universitat de València, Valencia, Spain

**Keywords:** depression, psychometric properties, impairment, severity, online

## Abstract

**Background:** The Overall Depression Severity and Impairment Scale (ODSIS) is a self-report scale designed to evaluate the severity and functional impairment associated with depression.

**Objective:** This study evaluated the psychometric properties of the online version of the ODSIS in Spanish outpatients with depression and anxiety disorders.

**Method:** Patients with a main diagnosis of a depressive (*n* = 283) or anxiety disorder (*n* = 191) and a mean age of 38.15 (*SD* = 12.06) were evaluated with a clinical diagnostic interview and measures assessing depression, anxiety, positive and negative affect, and quality of life. Factorial structure, internal consistency, convergent, and discriminant validity and cutoff scores were analyzed.

**Results:** Consistent with previous validations of the instrument, Confirmatory Factor Analysis showed a unidimensional factor structure. Furthermore, the results obtained supported the internal consistency and construct validity of the ODSIS scores. A score of 5 was found to meet the criteria used in this study for the optimal cutoff score.

**Conclusion:** The results obtained in this study show that the Spanish version of the ODSIS delivered online is an adequate tool to assess the depression-related severity and impairment in a brief and easy fashion.

## Introduction

Among the emotional disorders, the most prevalent lifetime syndrome is a major depressive episode (1, 2). It is among the most common reasons for consulting a general practitioner (3–5). This happens not only in adult's population, but also among young people the major depressive disorder (MDD) is considered one of the leading causes of disability (6–9). In Spain, depression is the most pervasive mental disorder, with a 12-month prevalence estimated at 3.9%, and a lifetime prevalence estimated at 10.5% (6). To the epidemiological magnitude of this disease, it is important to add its special tendency toward chronicity (7) and its high rate of comorbidity (10), especially with anxiety disorders (11). Moreover, research has shown that depression is associated with significant functional impairment, with poorer quality of life, disability, and important costs (personal, social, and economic) (2, 10, 12). Furthermore, regarding the functioning domains, depressive symptoms have stronger associations with role limitations due to vitality, mental health, physical and emotional, and social functioning problems than anxiety symptoms (2). In view of these data, there is a need to develop screening strategies for the emotional and behavioral symptoms related to MDD (6, 13). Furthermore, because depressive symptoms are accompanied by significant distress and impairment, it is important to capture the severity of the impairment and assess it in research and clinical settings.

With the increasing interest in the identification and dissemination of evidence-based treatments for psychological problems, the need for evidence-based assessment strategies is greater than ever before. It is crucial for researchers to develop assessment instruments with good psychometric properties resulting in their reliable evaluation. Furthermore, because research on Internet-based interventions has grown exponentially in recent years, it is also necessary to validate online assessment scales with good psychometric properties (14, 15).

As mentioned above, depression is among the most prevalent and disabling psychological disorders worldwide. Currently, a variety of well-validated measures exist to assess different aspects of depression, such as the Patient Health Questionnaire-9 [PHQ-9; (16)], the Center for Epidemiological Studies Depression Scale [CES-D; (17)], and the Beck Depression Inventory-II [BDI-II; (18)]. Nevertheless, the existing depression scales are lengthy and largely focused on the frequency of cognitive, affective, or somatic symptoms related to depression, rather than overall severity and the resulting impairment.

The literature shows that it is essential to assess the severity and impairment associated with depression because these factors influence whether or not individuals may benefit from treatment (19). Thus, because depression usually leads to important impairments, it is necessary to measure this aspect (i.e., how depression affects a patient's daily life and interpersonal relationships) in order to determine the effectiveness of an intervention (18). Indeed, according to the Diagnostic and Statistical Manual of Mental Disorders, Fifth Edition (DSM-5) (20), functional impairment is a basic criterion in depressive disorders.

The Overall Depression Severity and Impairment Scale (ODSIS) is a 5-item assessment instrument to evaluate severity and functional impairment due to depressive symptoms, such as the frequency and intensity of depression and its interference with work, school, social life, and relationships. Furthermore, it also assesses the impairment caused by depression-related loss of interest and difficulty engaging in activities (20). All of these questions are rated on a Likert-type scale ranging from 0 to 4 and referring to an “in the past week” time frame, with higher scores indicating greater depression-related severity and impairment.

To date, only two validation studies of the ODSIS have been published. The first validation of the ODSIS was conducted in a North American sample (19), whereas the second was performed in a non-western sample (21). Both validations showed good psychometric properties, such as optimal internal consistency and adequate convergent and discriminant validity. Among the advantages of the ODSIS, the authors highlighted its accuracy in detecting depressive disorders, in spite of its conciseness, and the fact that the scale focuses on general functional impairment rather than specific symptoms (19, 21).

To the best of our knowledge, no study has evaluated the psychometric properties of the online version of the ODSIS in a sample of adults with depression and anxiety disorders in the Spanish population. In addition, although an online validation of the ODSIS has already been carried out (21), the diagnoses of the participants in this study were based on their own self-reports, rather than well-validated instruments such as diagnostic interviews, as in the present study. Specifically, the participants were asked whether they were “diagnosed as having major depressive disorder and being treated for the problem in a medical setting” [(21), p. 4].

In a previous study published by the current authors, the psychometric properties of the Overall Anxiety Severity and Impairment Scale (OASIS) were analyzed (22). Similarly to the ODSIS, the OASIS is a 5-item scale for the assessment of the severity and impairment associated with anxiety. Therefore, using the same pool of individuals, in this study, we explored the psychometric properties of the ODSIS in two clinical subsamples of Spanish individuals with emotional disorders: a subsample with a principal diagnosis of depression (*n* = 283) and a subsample with a principal diagnosis of anxiety (*n* = 191). Thus, we formulated the following objectives: (a) to examine how the scale performs in patients with depressive vs. anxiety disorders; (b) to examine the scale's internal structure, reliability, and validity of the test scores; and (c) to obtain a cutoff score.

## Methods

### Procedure

The procedure in the present study was the same than in the recently published study regarding the Spanish validation of the OASIS (22). Before starting the study, a translation of the scale from English to Spanish was performed using the procedure described in González-Robles et al. (22). Individuals who were waiting to receive an online psychological treatment at the Emotional Disorders Clinic (Universitat Jaume I) were informed that a research study was being conducted, and they were invited to participate. Participants who agreed to participate, that were 18 years old, signed an informed, written consent, and met Diagnostic and Statistical Manual of Mental Disorders Fourth Edition (DSM-IV-TR) (23) criteria for a principal diagnosis of an emotional disorder (i.e., anxiety or depressive disorder) were considered for the study. DSM-IV-TR diagnoses were ascertained using the MINI-International Neuropsychiatric Interview (MINI) (24) by doctoral students who had been previously trained in the use of the diagnostic interview. Following the administration of the interview, the participants completed an online battery of questionnaires on depression, anxiety, positive and negative affect, and quality of life. All these measurement instruments are described in the Instruments section. Ethical approval for the research study was obtained from the Ethics Committee of Universitat Jaume I.

### Participants

A total of 474 individuals participated in the study. As aforementioned, we included the same participants that took part in the recent Spanish validation of the OASIS. However, the number of participants in the present study is lower because a portion of the sample did not answer the ODSIS. The average age was 38.15 (*SD* = 12.06; range: 18–68 years), and women made up the majority of the sample (*n* = 342; 72.15%). Most participants were married or had partners (*n* = 242; 51.05%), and about half had completed university studies (*n* = 235; 49.58%). In all, 283 participants had a main diagnosis of a depressive disorder (i.e., major depressive disorder, dysthymic disorder, depression not otherwise specified), and 191 had a main diagnosis of an anxiety disorder. The sample had a high comorbidity rate, with more than half of the participants (57.81%) presenting with at least one comorbid anxiety and/or depressive disorder. Table 1 summarizes participants' sociodemographic and clinical characteristics. Furthermore, Supplementary Datasheet 1 has the demographic and clinical data.

**Table 1:** Sociodemographic and clinical features of the sample (*N* = 474).

**Age in years, Mean (*SD*)**	**38.15 (12.06)**
**SEX**, ***n*** **(%)**	
Female	342 (72.2)
Male	132 (27.8)
**RELATIONSHIP STATUS**, ***n*** **(%)**	
Single	174 (36.7)
Married/de facto	242 (51.1)
Divorced	52 (11.0)
Widowed	6 (1.3)
**EDUCATION LEVEL**, ***n*** **(%)**	
Basic	81 (17.1)
Medium	158 (33.3)
University	235 (49.6)
**PRINCIPAL DIAGNOSIS**, ***n*** **(%)**	
Major depressive disorder (MDD)	273 (57.6)
Generalized anxiety disorder (GAD)	76 (16.0)
Social anxiety disorder (SAD)	44 (9.3)
Panic disorder/agoraphobia (PD/AG)	43 (9.1)
Obsessive-compulsive disorder (OCD)	15 (3.2)
Anxiety NOS	10 (2.1)
Dysthymic disorder	8 (1.7)
Posttraumatic stress disorder (PTSD)	3 (0.6)
Depression NOS	2 (0.4)
**NUMBER OF COMORBID DISORDERS**, ***n*** **(%)**	
0	200 (42.2)
1	184 (38.8)
2	69 (14.6)
≥ 3	21 (4.4)
**SYMPTOM SEVERITY, MEAN (SD)**	
ODSIS	7.83 (4.90)
BDI-II	23.08 (10.97)
BAI	19.95 (12.01)
OASIS	8.73 (4.22)
PANAS-N	25.91 (8.00)
PANAS-P	20.94 (7.23)
QLI	4.92 (1.67)

*ODSIS, Overall Depression Severity and Impairment Scale; BDI-II, Beck Depression Inventory-II; BAI, Beck Anxiety Inventory; OASIS, Overall Anxiety Severity and Impairment Scale; PANAS-N, Positive and Negative Affect Schedule-Negative Affect; PANAS-P, Positive and Negative Affect Schedule-Positive Affect; QLI, Multidimensional Quality of Life Questionnaire*.

### Instruments

#### Diagnostic Interview

##### Mini-international neuropsychiatric interview (24)

The MINI is a brief, structured diagnostic psychiatric interview that yields DSM-IV and ICD-10 diagnoses. The interview has shown excellent interrater and test-retest reliability. The Spanish validation of the MINI was employed in this study (25).

#### Self-Reported Questionnaires

##### Overall depression severity and impairment scale (ODSIS) (19)

The ODSIS is a brief self-reported scale with 5 items that assess the severity and functional impairment associated with depressive symptoms. Items are coded on a 5-point scale (0–4). The sum of the scores is used to obtain the total score, which can be a maximum of 20. The measure has shown excellent internal consistency (α = 0.94 in an outpatient sample, 0.92 in a community sample, and 0.91 in a student sample) and good convergent/discriminant validity (21). In the present study, Cronbach's alpha for the ODSIS was excellent (α = 0.93).

##### Beck depression inventory (BDI-II) (18)

The BDI-II is one of the most widely used scales for the assessment of depressive symptoms. The scale is made up of 21 items, rated from 0 to 3, about the different symptoms characterizing major depression disorder. The sum of the scores is used to obtain the total score, which can be a maximum of 63 points. The BDI-II has shown good internal consistency (α = 0.76–0.95). The Spanish validation has also demonstrated high internal consistency for both general (α = 0.87) and clinical populations (α = 0.89) (26). In the current study, the internal consistency of the BDI-II was excellent (α = 0.90).

##### Beck anxiety inventory (BAI) (27)

The BAI is a 21-item self-reported questionnaire that evaluates anxiety symptoms in a past week timeframe. Each item is rated from 0 (“not at all”) to 3 (“severely”), and scores can range between 0 and 63 points. The BAI has shown good to excellent internal consistency in previous validations (Cronbach's alpha between 0.85 and 0.94), as well as adequate convergent and divergent validity (28). In the current study, Cronbach's alpha for the BAI was excellent (α = 0.91).

##### Overall anxiety severity and impairment scale (OASIS) (29)

The OASIS is a 5-item self-reported scale that assesses the severity, impairment (work and social), and behavioral avoidance caused by anxiety symptoms. Previous studies have shown good internal consistency (α = 0.80), test-retest reliability, and convergent and discriminant validity (29–31). The Spanish version of the OASIS showed good internal consistency (α = 0.86), convergent and discriminant validity, as well as sensitivity to change (16). Cronbach's alpha for the OASIS in the present study was good (α = 0.86).

##### Positive and negative affect schedule (PANAS) (32)

The PANAS is a scale that evaluates two independent dimensions of affect: positive affect (PANAS-P) and negative affect (PANAS-N). Each subscale is made up of 10 items with descriptors (e.g., “scared” or “anxious” for the PANAS-N; “active” or “inspired” for the PANAS-P), and individuals have to select the degree to which they have experienced each of them in the past week on a 5-point Likert scale, with total scores ranging from 10 to 50 points. This measure has shown excellent convergent and divergent validity, as well as high reliability indexes (32, 33). In this study, Cronbach's alphas for this instrument were good to excellent (α = 0.88 for the PANAS-N and α = 0.93 for the PANAS-P).

##### Multidimensional quality of life questionnaire (QLI) (34)

The QLI is a 10-item self-reported scale assessing the following areas regarding quality of life: psychological well-being, physical well-being, self-care and independent functioning, occupational functioning, interpersonal functioning, emotional and social support, community and service support, self-realization, spiritual satisfaction, and a global assessment of quality of life. Previous psychometric evaluations of the QLI have found good internal consistency and test-retest reliability (34, 35). In the current study, Cronbach's alpha was good (α = 0.89).

#### Data Analysis

We followed the same statistical procedures for the analyses of data as in González-Robles et al. (22). First, descriptive statistics for the depression and anxiety subsamples were calculated for the ODSIS. In addition, an analysis of outliers was conducted with the ODSIS and BDI-II scores, such that a score was considered an outlier if it deviated from the median more than 1.5 times the interquartile range (36). Second, one-way ANOVAs were performed to compare the total scores on the ODSIS according to gender, relationship status, studies and principal diagnosis, and number of comorbid diagnoses. Bivariate correlations were calculated to analyze whether there was any association between age and ODSIS scores. In addition, reliability was analyzed by calculating internal consistency indexes (Cronbach's alpha) for the five items on the ODSIS.

As in González-Robles et al. (22), the latent structure of the ODSIS was analyzed using Confirmatory Factor Analysis (CFA), a method based on Classical Test Theory (CTT) (37). Maximum likelihood estimation and robust corrections were used to estimate the CFA models, given the scale's non-normality and five-point response scale. Full Information Maximum Likelihood was employed to handle missing data. Following the validation by Bentley et al. (19), a unidimensional factor structure was tested as the basis for the CFA model. A number of criteria were used to assess model fit: the chi-square test (χ^2^), the comparative fitness index (CFI), standardized root mean residuals (SRMR), and the root mean square error or approximates (RMSEA). The following cutoff scores were used to determine good fit: CFI and TLI above 0.90 (better if above 0.95) and RMSEA below.08 (38–40).

To examine construct validity of the test scores, the ODSIS was correlated with measures of anxiety (OASIS, BAI), depression (BDI-II), positive and negative affect (PANAS-P and PANAS-N) and quality of life (QLI). To interpret the correlation values, Cohen's (41) recommendations were followed: effect sizes between 0.10 and 0.30 are small; between 0.30 and 0.50 are medium; and 0.50 or above are large.

To assess the sensitivity and specificity of the ODSIS scores in detecting depressive symptoms, participants were classified according to published cutoffs of the BDI-II, so that patients with scores below 14 were considered to do not have zero or minimal depressive symptoms, while those with scores equal or above 14 to have mild, moderate or severe depressive symptoms (18). To examine the precision of the ODSIS scores in detecting cases with and without depressive symptoms, the receiver operating characteristic (ROC) curve and the area under the curve (*AUC*) were calculated. A 95% confidence interval for the *AUC* and its statistical significance were also calculated (42). In addition, Sensitivity, Specificity, Positive Predictive Value (*PPV*), and Negative Predictive Value (*NPV*) were obtained for each ODSIS cutoff point around the range of scores. In order to identify the optimal cutoff point on ODSIS scores to offer the best balance between sensitivity and specificity, four different methods were applied to each ODSIS cutoff point: Youden index (J), Index of Union (IU), Closest to (0, 1) Criterion, and Concordance Probability Method (CZ) (43). The ODSIS score that met the four criteria, or most of them, was selected as the optimal cutoff point. A detailed description of the statistical procedures followed in this study can be found in González-Robles et al. (22).

The following software was employed for the analyses. CFA was calculated using the software EQS (version 6). The sensitivity, specificity, PPV, NPV, and their 95% confidence intervals were calculated using the web application http://vassarstats.net/clin1.html. The remaining analyses were performed through the software SPSS Statistics, version 22.0.

## Results

### Preliminary Analyses

The mean ODSIS score was 7.83 (*SD* = 4.90) for the total sample (*n* = 474), 7.94 (*SD* = 4.98) for females (*n* = 342), and 7.53 (*SD* = 4.67) for the male participants (*n* = 132). Means and standard deviations for each item and the total score on the ODSIS are shown in Table 2 for both the depressive and anxiety disorder samples. The analyses of outliers for the ODSIS and the BDI-II with interquartile range did not show the presence of scores that can be considered as outliers. No significant differences were found based on gender (*d* = 0.08) and marital status (*Eta*^2^ = 0.012). A slight but significant correlation between age and the ODSIS scores was observed (*r* = 0.09, *p* < 0.05). The scores on the ODSIS in the depression sample were significantly higher than those in the anxiety sample [*F*_(1, 472)_ = 22.76; *p* < 0.001; *d* = 0.45]. Additionally, statistically significant differences in the ODSIS scores were found as a function of education level [*F*_(2, 471)_ = 5.43; *p* < 0.01; *Eta*^2^ = 0.023]. Sidak's *post-hoc* tests showed significant differences in depression levels in patients with medium studies compared to those with university studies, with lower levels of depression in the latter (*p* < 0.01). Significant differences were also found depending on the principal diagnosis [*F*_(8, 465)_ = 4.23; *p* < 0.001; *Eta*^2^ = 0.068] and number of comorbid disorders [*F*_(3, 470)_ = 5.71; *p* < 0.01; *Eta*^2^ = 0.035]. Sidak's *post-hoc* tests showed that patients with MDD as the principal diagnosis were significantly more depressed than patients with PD/AG as the principal diagnosis (*p* < 0.01). Additionally, *post-hoc* tests revealed that patients with 3 or more comorbid diagnoses had significantly higher levels of depression than those without any comorbid disorder (*p* < 0.05).

**Table 2:** Descriptive statistics for each item and the total score on the ODSIS in depression and anxiety samples.

	**Depression (*****n*** **= 283)**		**Anxiety (*****n*** **= 191)**	
	**M**	**SD**	**M**	**SD**
Item 1	1.80	1.03	1.43	1.09
Item 2	1.67	0.95	1.30	1.02
Item 3	1.88	1.16	1.40	1.17
Item 4	1.65	1.09	1.24	1.30
Item 5	1.69	1.11	1.25	1.18
Total score	8.69	4.68	6.55	4.94

### Factor Structure

Adequate model fit was found for a single-factor model, with the following values: χ^2^_(4)_ = 16.08, *p* > 0.001; SRMR = 0.03, RMSEA = 0.08, 90% CI (0.04, 0.12); CFI = 0.99. All items were strongly related to this factor, as shown by the factor loadings, with values that ranged from 0.84 to 0.88.

### Internal Consistency

Cronbach's alpha for the five items on the ODSIS was 0.92. Table 3 displays the results for Cronbach's alpha when omitting items, corrected correlations between each item, and the total score, and correlations between the five items of the ODSIS. The outcomes indicate good internal consistency of the ODSIS scores that would not be increased by excluding any item.

**Table 3:** Cronbach's alpha if item is deleted, corrected item-total score correlation, and correlations between items.

	**Cronbach's alpha if item deleted**	**Corrected item-total correlation**	**Correlations between items**				
			**Item 1**	**Item 2**	**Item 3**	**Item 4**	**Item 5**
Item 1	0.906	0.800	1				
Item 2	0.907	0.801	0.776^*^	1			
Item 3	0.898	0.836	0.722^*^	0.707^*^	1		
Item 4	0.912	0.771	0.659^*^	0.677^*^	0.723^*^	1	
Item 5	0.905	0.803	0.686^*^	0.683^*^	0.780^*^	0.687^*^	1

* *All correlations were statistically significant at p < 0.01 (2-tailed)*.

### Convergent and Discriminant Validity

Table 4 summarizes the correlation coefficients between the ODSIS and convergent and discriminant measures. A large and positive correlation between the ODSIS and the BDI-II was expected. A positive but medium correlation was expected between the ODSIS and the anxiety measures (OASIS and BAI). Furthermore, given that the PANAS and the BDI-II assess different but related constructs (44), a positive but medium correlation between the ODSIS and the PANAS-N, and a negative but medium correlation between the ODSIS and the PANAS-P were expected. Finally, because quality of life is highly dependent on psychological well-being (45), a negative, medium to high correlation between the ODSIS and the QLI was predicted. Weaker correlations between the ODSIS and measures of anxiety, positive/negative affect, and quality of life than with depression measures were interpreted as evidence for discriminant validity of the test scores.

**Table 4:** Correlations of the ODSIS with convergent validity measures.

	**ODSIS**	**BDI-II**	**BAI**	**OASIS**	**PANAS-N**	**PANAS-P**	**QLI**
ODSIS	1	0.67^*^	0.37^*^	0.47^*^	0.49^*^	−0.56^*^	−0.70^*^
BDI-II		1	0.44^*^	0.47^*^	0.55^*^	−0.54^*^	−0.77^*^
BAI			1	0.62^*^	0.55^*^	−0.25^*^	−0.41^*^
OASIS				1	0.42^*^	−0.29^*^	−0.56^*^
PANAS-N					1	−0.27^*^	−0.47^*^
PANAS-P						1	0.71^*^
QLI							1

* *All correlations were statistically significant at p < 0.01 (2-tailed)*.

*ODSIS, Overall Depression Severity and Impairment Scale; BDI-II, Beck Depression Inventory-II; BAI, Beck Anxiety Inventory; OASIS, Overall Anxiety Severity and Impairment Scale; PANAS-N, Positive and Negative Affect Schedule-Negative Affect; PANAS-P, Positive and Negative Affect Schedule-Positive Affect; QLI, Multidimensional Quality of Life Questionnaire*.

The ODSIS significantly correlated with all the measures. A positive and large correlation between the ODSIS and the BDI-II was found, as expected (*r* = 0.67, *p* < 0.01). Furthermore, medium and positive correlations were observed between the ODSIS and the anxiety measures (OASIS and BAI), with values ranging between 0.37 and 0.47 (*p* < 0.01). In addition, the ODSIS correlated negatively but highly with the PANAS-P (*r* = −0.56, *p* < 0.01), and positively but moderately with the PANAS-N (*r* = 0.49, *p* < 0.01). Finally, a large and positive correlation was observed between the ODSIS and the QLI (*r* = 0.70, *p* < 0.01).

### ROC Analysis

**Figure 1:**
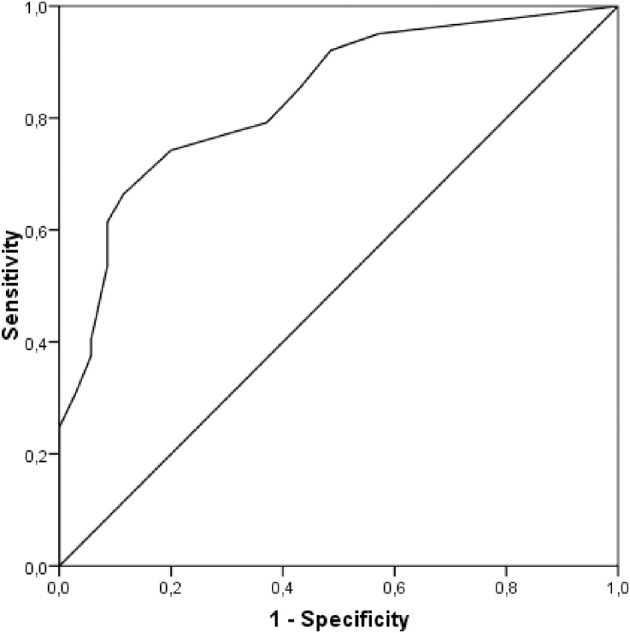
ROC curve.

A ROC curve was calculated in the sample of 136 participants. Not all the participants of the sample completed the BDI-II measure at pre-test. For this reason, we did the analysis with 136 participants. The scores on the BDI-II were used as criterion, such that participants with scores ≥ 14 on the BDI-II were classified as suffering from mild, moderate or severe depressive symptoms, whereas participants with scores below 14 were classified as suffering from zero or minimal depressive symptoms (18). The AUC obtained was 0.84 (95%CI: 0.76 and 0.91) and reached statistical significance (*p* < 0.001). Figure 1 displays a graphical representation of the ROC curve. This AUC can be interpreted as indicating that there was a 0.84 probability of randomly selecting a participant from the mild, moderate or severe depressive symptoms group (i.e., with a BDI-II score ≥ 14) with an ODSIS score higher than that of any other participant, also randomly selected, from the zero or minimal depressive symptoms group (i.e., with BDI-II score < 14). An AUC = 0.84 can be interpreted as reflecting moderate precision from a clinical point of view. Therefore, the precision of the ODSIS scores in detecting depressive symptoms can be considered to have a moderate magnitude.

Table 5 presents the sensitivity, specificity, PPV, and NPV obtained with the ODSIS scores for the cutoff point ≥ 14 on the BDI-II. Table 5 also shows the results obtained from applying four methods to select the optimal cutoff point for the ODSIS scores [Youden index, J, Index of Union, IU, the Closest to (0, 1) Criteria, ER, and the Concordance Probability Method, CZ]. Out of the four methods, the ODSIS score = 5 met three of the four criteria (IU, ER, and CZ criteria), and on the Youden index, this score obtained the second-best value, very close to the first one. Therefore, the optimal selected cutoff point on the ODSIS was 5 (i.e., ODSIS scores ≥ 5 indicating depressive symptoms). For this cutoff point, sensitivity was 0.74 (95%CI: 0.64 and 0.82), whereas specificity was 0.80 (95%CI: 0.63 and 0.91). PPV was 0.92 (95%CI: 0.83 and 0.97), and NPV was 0.52 (95%CI: 0.38 and 0.66; see bold values provided in Table 5).

**Table 5:** Statistics to assess the diagnostic accuracy of the ODSIS scores.

**ODSIS score**	***Se***	***Sp***	***PPV***	***NPV***	***J***	***IU***	***ER***	***CZ***
1	0.950	0.429	0.827	0.750	0.379	0.521	0.573	0.408
2	0.921	0.514	0.845	0.692	0.435	0.407	0.492	0.473
3	0.851	0.571	0.851	0.571	0.422	0.280	0.454	0.486
4	0.792	0.629	0.860	0.512	0.421	0.253	0.425	0.498
**5**	**0.743**	**0.800**	**0.915**	**0.519**	0.543	**0.131**	**0.326**	**0.594**
6	0.663	0.886	0.944	0.477	**0.549**	0.223	0.356	0.587
7	0.614	0.914	0.954	0.451	0.528	0.300	0.395	0.561
8	0.535	0.914	0.947	0.405	0.449	0.379	0.473	0.489
9	0.406	0.943	0.953	0.355	0.349	0.537	0.597	0.383
10	0.376	0.943	0.950	0.344	0.319	0.567	0.627	0.355
11	0.307	0.971	0.969	0.327	0.278	0.664	0.694	0.298
12	0.248	1	1	0.315	0.248	0.752	0.752	0.248
13	0.218	1	1	0.307	0.218	0.782	0.782	0.218
14	0.149	1	1	0.289	0.149	0.851	0.851	0.149
15	0.109	1	1	0.280	0.109	0.891	0.891	0.109
16	0.099	1	1	0.278	0.099	0.901	0.901	0.099
17	0.040	1	1	0.266	0.040	0.960	0.960	0.040
18	0	1	NA	0.257	0	1	1	0

*Se, Sensitivity; Sp, Specificity; PPV, Positive Predictive Value; NPV, Negative Predictive Value; J, Youden index; IU, Index of Union; ER, Closest to (0, 1) Criteria; CZ, Concordance Probability Method; NA, Not Applicable*.

## Discussion

The aim of this study was to analyze the psychometric properties of the ODSIS administered online in a Spanish sample of patients with depression and anxiety disorders. The literature has shown the high comorbidity rates between depressive and anxiety disorders (10); therefore, it is important to explore how the scale performs in both diagnostic groups. To our knowledge, this is the first study to evaluate its psychometric properties in a Spanish clinical sample using the online version of the instrument. This study evaluated the reliability and construct validity of the test scores, latent structure, and cutoff scores of the ODSIS.

First, preliminary analyses showed no significant differences based on gender or marital status. However, patients with medium studies were significantly more depressed than those with university studies. In addition, a slight but significant correlation was observed between age and the ODSIS scores. By contrast, the original validation did not find significant differences with regard to any sociodemographic or clinical variable (19). Second, the scores on the ODSIS in the depression sample were significantly higher than those in the anxiety sample. These results suggest that, in this study, the ODSIS was able to discriminate between depressive and anxiety disorders, a property of the instrument that is also found in the validation by Bentley et al. (19). Furthermore, patients with more comorbid diagnoses had significantly higher levels of depression than those without any comorbid disorder. These data are consistent with the literature showing strong associations between comorbidity and severity (10). These results demonstrate how the scale performs in different populations, which is essential, given the importance of developing brief measures that are applicable across diverse clinical and research settings with distinct samples. Because it is a very brief instrument, the ODSIS is expected to be quite suitable for use in different settings and contexts, such as research, primary care, clinical routine monitoring, and epidemiological studies.

With regard to reliability, the ODSIS demonstrated excellent internal consistency in the Spanish clinical sample (alpha = 0.93), consistent with previous validation studies (19, 21). Finally, also, as in previous validations of the instrument (19, 21), confirmatory factor analysis revealed a unidimensional factor structure with strong factor loadings for all the items.

Regarding cutoff scores, a cutoff point of 5 was found to meet three of the four criteria used to select the optimal cutoff point [i.e., Index of Union, Closest to (0, 1) Criterion, and Concordance Probability Method]. Thus, these outcomes suggest that this score can be used as a cutoff point to discriminate patients with clinical depressive symptoms from those with no clinical depressive symptoms. These data might be useful, for instance, for selecting patients with depressive symptoms for clinical trials. The results obtained in this study using ROC analysis are not consistent with prior validations of the instrument in clinical populations because Bentley et al. (19) obtained a cutoff score of 8, and Ito et al. (21) provided a cutoff score of 11.There could be a number of reasons for these differences. For example, participants in this study were classified based on a cutoff score from a self-reported scale (BDI-II) [i.e., patients scoring ≥ 14 were classified as suffering from clinical levels of depression (mild, moderate or severe depressive symptoms), whereas those with scores under 14 were classified as not suffering from clinical levels of depression (zero or minimal depressive symptoms)], the samples had different characteristics (different clinical diagnoses, different countries, etc.), and there were differences in the mean ODSIS scores (7.83 for the Spanish clinical sample, 5.50 for the U.S. clinical sample, and 6.51 for the Japanese clinical sample examined here). These discrepancies might also account for the differences between the cutoff scores obtained in the present study and those from prior validations. Finally, the results by Bentley et al. (19) and Ito et al. (21) are not consistent with each other either. Thus, additional research is warranted to further explore how the ODSIS performs in detecting patients with clinical levels of depression.

The analysis of construct validity of the test scores yielded evidence for relation with all other variables. As anticipated, positive and relevant correlations were found between the ODSIS and measures of negative affect, depressive symptoms, and anxiety. Large and significant correlations between the ODSIS and the BDI-II were interpreted as evidence of convergent validity. Moreover, correlations between the ODSIS with anxiety (OASIS, BAI) and affect (PANAS-P and PANAS-N) of lower magnitude than with depression (BDI) were interpreted as evidence of discriminant validity. Additionally, the positive and large correlation observed between the ODSIS and the QLI suggests the strong association between high levels of depression and poorer quality of life. Overall, the results obtained in this study support the construct validity of the ODSIS scores.

The strengths of this study are highlighted in the following lines. First, this is the first study to evaluate the psychometric properties of the ODSIS delivered online in a Spanish clinical population. In clinical settings, the time that the clinicians have available to assess patients is usually very limited. Brief instruments like the ODSIS can help in the evaluation of the severity and impairment caused by depression, making this task simpler. Second, as mentioned above, depression is characterized by high comorbidity rates (10), especially with the anxiety disorders (44), and the present study presents data about how the ODSIS works in patients, not only those with depression, but also those with an anxiety disorder as a main diagnosis. Indeed, the heterogeneity of the sample (patients with a range of anxiety and depressive disorders) can help to increase the generalizability of the results obtained in this study. Third, this study provides further support for the literature on the ODSIS, suggesting that it can be used in an online format without compromising its psychometric properties, coinciding with the validation by (21). Currently, as the field of Internet interventions expands, and because the psychometric properties of the same instrument may differ depending on the way it is administered (i.e., pencil and paper vs. online) (46), the need for studies to validate online versions of well-established traditional scales is clear.

## Limitations

This study has limitations that should be mentioned. First, even though the BDI-II is a well-established measure and one of the more widespread scales for the assessment of depression (47, 48), we did not follow the optimum approach for the calculation of the ROC curve because the classification of subjects was based on a cutoff from a scale (BDI-II) rather than a group of healthy control individuals. Therefore, caution should be taken when using the cutoff score obtained in this study. Second, the convergent validity of the ODSIS was evaluated in relation to only one measure of depression (BDI-II). Although it would have been desirable to include other depression scales to more in depth explore the convergent validity of the ODSIS scores, this was not possible because all the participants in this study were referred from clinical trials where the selection of assessment instruments was already pre-determined. Finally, we did not evaluate the test-retest reliability of the ODSIS.

## Conclusions and Future Directions

To conclude, the results obtained in this study support the internal consistency, factorial structure, and construct validity of the ODSIS delivered online in a Spanish clinical sample with a range of depressive and anxiety disorders, consistent with those obtained in prior validations of the scale (16, 18).

Future validations of the ODSIS in Spanish clinical patients are warranted to more precisely explore cutoff scores in a sample of healthy control individuals. In addition, because there is disparity between the cutoff scores obtained in this study (ODSIS = 5) and those obtained in previous validations of the ODSIS, more research is needed to replicate these findings. Another important aspect is that, given that the ODSIS focuses on depression-related severity and impairment, main targets of any psychological or psychiatric intervention, future studies should analyze the scale's sensitivity to change, i.e., how the scale performs as a treatment outcome measure. Finally, although the sample used in this study was rather heterogeneous, it was restricted to patients with depressive and anxiety disorders. Therefore, further research is warranted to analyze the validity and reliability of the ODSIS in more severe patients (e.g., bipolar or psychotic disorders).

## Ethics Statement

This study was carried out in accordance with the recommendations of Ethics Committee of Jaume I University with written informed consent from all subjects. All subjects gave written informed consent in accordance with the Declaration of Helsinki. The protocol was approved by the Ethics Committee of Jaume I University.

## Author Contributions

AM drafted the original manuscript and participated in the conceptualization and methodology. AG-R was involved in the conceptualization, writing and reviewing the manuscript. CM and AD-G were involved in the data curation and participated in writing the original draft, GM collaborated in the data curation and methology of the study. AG-P, JB-L, and SQ were involved in the conceptualization, supervision, and the revision of the manuscript. RB and CB were involved in the conceptualization, funding acquisition, supervision, writing, and review.

### Conflict of Interest Statement

The authors declare that the research was conducted in the absence of any commercial or financial relationships that could be construed as a potential conflict of interest.

## Supplementary Material

Supplementary Datasheet 1 Database ODSIS: demographic and clinical data.

## Abbreviations

AUC: area under the curve
BAI: Beck Anxiety Inventory
BDI-II: Beck Depression Inventory-II
CES-D: Center for Epidemiological Studies Depression Scale
CFA: Confirmatory Factor Analysis
CFI: comparative fitness index
CTT: Classical Test Theory
CZ: Criterion and Concordance Probability Method
DSM-IV-TR: Statistical Manual of Mental Disorders Fourth Edition
DSM-5: Diagnostic and Statistical Manual of Mental Disorders, Fifth Edition
GAD: Generalized anxiety disorder
IU: Index of Union
J: Youden index
MINI: Mini-International Neuropsychiatric Interview
NPV: Negative Predictive Value
OASIS: Overall Anxiety Severity and Impairment Scale
ODSIS: Overall Depression Severity and Impairment Scale
OCD: Obsessive-compulsive disorder
PANAS: Positive and Negative Affect Schedule
PANAS-N: Positive and Negative Affect Schedule-Negative Affect
PANAS-P: Positive and Negative Affect Schedule-Positive Affect
PD/AG: Panic disorder/agoraphobia
PHQ-9: Patient Health Questionnaire-9
PPV: Sensitivity, Specificity, Positive Predictive Value
QLI: Multidimensional Quality of Life Questionnaire
RMSEA: root mean square error or approximates
SAD: Social anxiety disorder
ROC curve: receiver operating characteristic
SRMR: Standardized root mean residuals.

## References

[1] Haro JM Ayuso-Mateos JL Bitter I Demotes-Mainard J Leboyer M Lewis SW. ROAMER: roadmap for mental health research in Europe. Int J Methods Psychiatr Res. (2014) 23:1–14. 10.1002/mpr.1406 24375532 PMC6878332

[2] Kessler RC Petukhova M Sampson NA Zaslavsky AM Wittchen H-U. Twelve-month and lifetime prevalence and lifetime morbid risk of anxiety and mood disorders in the United States. Int J Methods Psychiatr Res. (2012) 21:169–84. 10.1002/mpr.1359 22865617 PMC4005415

[3] Cano-Vindel A Salguero JM Mae Wood C Dongil E Latorre J M La depresión en atención primaria: prevalencia, diagnóstico y tratamiento. Papeles Psicól. (2012) 33:2–11.

[4] Üstün TB Ayuso-Mateos JL Chatterji S Mathers C Murray CJ. Global burden of depressive disorders in the year 2000. Br J Psychiatry. (2004) 184:386–92. 10.1192/bjp.184.5.386 15123501

[5] World Health Organization Depression and Other Common Mental Disorders. CC BY-NC-SA 30 IGO. World Health Organization (2017) p. 1–22.

[6] Ortuño-Sierra J Aritio-Solana R Inchausti F De Luis EC Molina BL De Albéniz AP. Screening for depressive symptoms in adolescents at school: new validity evidences on the short form of the reynolds depression scale. PLoS ONE. (2017) 12:e0170950. 10.1371/journal.pone.0170950 28222193 PMC5319653

[7] Gore FM Bloem PJ Patton GC Ferguson J Joseph V Coffey C Global burden of disease in young people aged 10-24 years: a systematic analysis. Lancet. (2011) 18:2093–102. 10.1016/S0140-6736(11)60512-6 21652063

[8] Haro JM Palacín C Vilagut G Martínez M Bernal M Alonso GEJ Prevalencia trastornos mentales. Med Clin. (2006) 126:445–51. 10.1157/13086324 16620730

[9] Andrews G. Should depression be managed as a chronic disease? BMJ. (2001) 322:419. 10.1136/bmj.322.7283.419 11179166 PMC1119639

[10] Kessler RC Chiu WT Demler O Walters EE. Prevalence, severity, and comorbidity of 12-month DSM-IV disorders in the national comorbidity survey replication. Arch Gen Psychiatry. (2005) 62:617–27. 10.1001/archpsyc.62.6.617 15939839 PMC2847357

[11] Brown TA Campbell LA Lehman CL Grisham JR Mancill RB. Current and lifetime comorbidity of the DSM-IV anxiety and mood disorders in a large clinical sample. J Abnorm Psychol. (2001) 110:585–99. 10.1037/0021-843X.110.4.585 11727948

[12] Kessler RC Berglund P Demler O Jin R Koretz D Merikangas KR. The epidemiology of major depressive disorder: results from the national comorbidity survey replication (NCS-R). JAMA. (2003) 289:3095–105. 10.1001/jama.289.23.3095 12813115

[13] Ortuño-Sierra J Fonseca-Pedrero E Paíno M Aritio-Solana R Prevalencia de síntomas emocionales y comportamentales en adolescentes españoles. Rev Psiquiatr Salud Ment. (2014) 7:121–30. 10.1016/j.rpsm.2013.12.003 24530346

[14] Brenes GA. Anxiety, depression, and quality of life in primary care patients. Prim Care Companion J Clin Psychiatry. (2007) 9:437–43. 10.4088/PCC.v09n0606 18185823 PMC2139931

[15] van Ballegooijen W Riper H Cuijpers P van Oppen P Smit JH. Validation of online psychometric instruments for common mental health disorders: a systematic review. BMC Psychiatry. (2016) 16:45. 10.1186/s12888-016-0735-7 26915661 PMC4766750

[16] Kroenke K Spitzer RL Williams JB. The PHQ-9: validity of a brief depression severity measure. J Gen Intern Med. (2001) 16:606–13. 10.1046/j.1525-1497.2001.016009606.x 11556941 PMC1495268

[17] Radloff L The CES-D scale: a self-report depression. Appl Psychol Meas. (1977) 1:385–400. 10.1177/014662167700100306

[18] Beck AT Steer RA Brown GK Manual for the Beck Depression Inventory-II. San Antonio, TX: Psychological Corporation (1996).

[19] Bentley KH Gallagher MW Carl JR Barlow DH. Development and validation of the overall depression severity and impairment scale. Psychol Assess. (2014) 26:815–30. 10.1037/a0036216 24708078

[20] American Psychiatric Association Diagnostic and Statistical Manual of Mental Disorders (DSM-V) (Fifth Edition). Washington, DC: American Psychiatric Association (2013).

[21] Ito M Bentley KH Oe Y Nakajima S Fujisato H Kato N. Assessing depression related severity and functional impairment: the Overall Depression Severity and Impairment Scale (ODSIS). PLoS ONE. (2015) 10:1–14. 10.1371/journal.pone.0122969 25874558 PMC4395441

[22] González-Robles A Mira A Miguel C Molinari G Garcia-Palacios A Bretón-López J. A brief online transdiagnostic measure: psychometric properties of the Overall Anxiety Severity and Impairment Scale (OASIS) among Spanish patients with emotional disorders. PLoS ONE. (2018) 13:e0206516. 10.1371/journal.pone.0206516 30383797 PMC6211825

[23] American Psychiatric Association (ed). Diagnostic and Statistical Manual of Mental Disorders. 4th ed, text revision. Washington, DC: American Psychiatric Association (2000).

[24] Sheehan DV Lecrubier Y Sheehan KH Amorim P Janavs J Weiller E. The Mini-International Neuropsychiatric Interview (M.I.N.I.): the development and validation of a structured diagnostic psychiatric interview for DSM-IV and ICD-10. J Clin Psychiatry. (1998) 59(Suppl. 2):22–33. 9881538

[25] Ferrando L Bobes J Gibert J MINI. Mini International Neuropsychiatric Interview. Versión en Español 5.0.0 DSM-IV. Instrum Detección Orient Diagn. (2000) 2–26.

[26] Sanz Fernández J Navarro ME Vázquez Valverde C Adaptación española del inventario para la depresión de Beck-II: 1. Propiedades psicométricas en estudiantes universitarios. Anál Modif Conduct. (2003) 29:239–88.

[27] Steer RA Ranieri WF Beck AT Clark DA Further evidence for the validity of the beck anxiety inventory with psychiatric outpatients. J Anxiety Disord. (1993) 7:195–205. 10.1016/0887-6185(93)90002-3

[28] Magán I Sanz J García-Vera MP. Psychometric properties of a Spanish version of the Beck Anxiety Inventory (BAI) in general population. Span J Psychol. (2008) 11:626–40. 10.1017/S1138741600004637 18988448

[29] Campbell-Sills L Norman SB Craske MG Sullivan G Lang AJ Chavira DA. Validation of a brief measure of anxiety-related severity and impairment: the overall anxiety severity and impairment scale (OASIS). J Affect Disord. (2009) 112:92–101. 10.1016/j.jad.2008.03.014 18486238 PMC2629402

[30] Norman SB Hami Cissell S Means-Christensen AJ Stein MB. Development and validation of an overall anxiety severity and impairment scale (OASIS). Depress Anxiety. (2006) 23:245–9. 10.1002/da.20182 16688739

[31] Norman SB Campbell-Sills L Hitchcock CA Sullivan S Rochlin A Wilkins KC. Psychometrics of a brief measure of anxiety to detect severity and impairment: the overall anxiety severity and impairment scale (OASIS). J Psychiatr Res. (2011) 45:262–8. 10.1016/j.jpsychires.2010.06.011 20609450 PMC2970755

[32] Crawford JR Henry JD. The positive and negative affect schedule (PANAS): construct validity, measurement properties and normative data in a large non-clinical sample. Br J Clin Psychol. (2004) 43:245–65. 10.1348/0144665031752934 15333231

[33] Sandín B Chorot P Lostao L Joiner TE Santed ME Valiente R Escalas PANAS de afecto positivo y negativo: validación factorial y convergencia transcultural. Psicothema. (1999) 11:37–51.

[34] Mezzich JE Ruipérez MA Pérez C Yoon G Liu J Mahmud S. The Spanish version of the quality of life index: presentation and validation. J Nerv Ment Dis. (2000) 188:301–5. 10830568 10.1097/00005053-200005000-00008

[35] Mezzich JE Cohen NL Ruiperez MA Banzato CEM Zapata-Vega MI. The multicultural quality of life index: presentation and validation. J Eval Clin Pract. (2011) 17:357–64. 10.1111/j.1365-2753.2010.01609.x 21208350

[36] Behrens JT Principles and procedures of exploratory data analysis. Psychol Methods. (1997) 2:131 10.1037/1082-989X.2.2.131

[37] DeVellis RF. Classical test theory. Med Care. (2006) 44:S50–9. 10.1097/01.mlr.0000245426.10853.30 17060836

[38] Hu L Bentler PM Cutoff criteria for fit indexes in covariance structure analysis: conventional criteria versus new alternatives. Struct Equat Model Multidiscipl J. (1999) 6:1–55.

[39] Marsh HW Hau KT Wen Z In search of golden rule : comment on hypothesis testing approaches to setting cutoff value for fit indexes and danger in overgeneralizing hu and bentler's (1999) Finding. Struct Equ Model. 11:320–41.

[40] Yu CY Evaluating Cutoff Criteria of Model fit Indices for Latent Variable Models with Binary and Continuous Outcomes (Vol. 30). Los Angeles, CA: University of California (2002).

[41] Cohen J Statistical power analysis for the behavioral sciences, 2nd Edn. Hillsdale, NJ: Erlbaum (1988).

[42] Argimon JM Jiménez J Métodos de Investigaciónclínica y Epidemiológica. 4th ed. Barcelona: Elsevier España (2013).

[43] Unal I. Defining an optimal cut-point value in ROC analysis: an alternative approach. Comput Math Methods Med. (2017). 10.1155/2017/3762651 28642804 PMC5470053

[44] Werner-Seidler A Banks R Dunn BD Moulds ML. An investigation of the relationship between positive affect regulation and depression. Behav Res Ther. (2013) 51:46–56. 10.1016/j.brat.2012.11.001 23178678

[45] Greenspoon PJ Saklofske DH Toward an integration of subjective well-being and psychopathology. Soc Indic Res. (2001) 54:81–108. 10.1023/A:1007219227883

[46] Alfonsson S Maathz P Hursti T. Interformat reliability of digital psychiatric self-report questionnaires: a systematic review. J Med Internet Res. (2014) 16:e268. 10.2196/jmir.3395 25472463 PMC4275488

[47] Josephine K Josefine L Philipp D David E Harald B. Internet-and mobile-based depression interventions for people with diagnosed depression: a systematic review and meta-analysis. J Affect Disord. (2017) 223:28–40. 10.1016/j.jad.2017.07.021 28715726

[48] Karyotaki E Kleiboer A Smit F Turner DT Pastor AM Andersson G. Predictors of treatment dropout in self-guided web-based interventions for depression: an ‘individual patient data’ meta-analysis. Psychol Med. (2015) 45:2717–2726. 10.1017/S0033291715000665 25881626

